# Is there a role for perfusion imaging in assessing treatment response following ablative therapy of small renal masses—A systematic review^☆^

**DOI:** 10.1016/j.ejro.2018.07.002

**Published:** 2018-07-24

**Authors:** S.J. Withey, J. Gariani, K. Reddy, D. Prezzi, C. Kelly-Morland, S. Ilyas, A. Adam, V. Goh

**Affiliations:** a Department of Radiology, Guy’s and St Thomas’ NHS Foundation Trust, London, United Kingdom; b Cancer Imaging, Division of Imaging Sciences and Biomedical Engineering, King’s College London, United Kingdom

**Keywords:** Ablation, Renal, Radiofrequency, Cryoablation, Perfusion CT, Dual energy CT, Contrast-enhanced MRI, Response assessment

## Abstract

**Aims:**

Ablation therapies are an innovative nephron-sparing alternative to radical nephrectomy for early stage renal cancers, although determination of treatment success is challenging. We aimed to undertake a systematic review of the literature to determine whether assessment of tumour perfusion may improve response assessment or alter clinical management when compared to standard imaging.

**Material and Methods:**

Two radiologists performed independent primary literature searches for perfusion imaging in response assessment following ablative therapies (radiofrequency ablation and cryotherapy) focused on renal tumours.

**Results:**

5 of 795 articles were eligible, totaling 110 patients. The study designs were heterogeneous with different imaging techniques, perfusion calculations, reference standard and follow-up periods. All studies found lower perfusion following treatment, with a return of ‘high grade’ perfusion in the 7/110 patients with residual or recurrent tumour. One study found perfusion curves were different between successfully ablated regions and residual tumour.

**Conclusions:**

Studies were limited by small sample size and heterogeneous methodology. No studies have investigated the impact of perfusion imaging on management. This review highlights the current lack of evidence for perfusion imaging in response assessment following renal ablation, however it suggests that there may be a future role. Further prospective research is required to address this.

## 1 Introduction

In 2014, there were 12,523 new cases of renal cancer in the UK [1], a number increased by 78% since the 1990s [1]. This can be partly attributed to the increased use of cross-sectional imaging and the consequent incidental finding of small, localised renal masses. As incidental small renal masses have been shown to be generally of lower grade and associated with longer disease-free survival than their symptomatic counterparts [2], nephron-sparing treatments have become preferable to conserve renal function. The surgical gold-standard is now considered to be partial nephrectomy (PN) [3].

An alternative to PN is ablative therapy either using high (radiofrequency ablation, RFA; microwave ablation) or low temperatures (cryotherapy). These techniques are particularly suited to patients with co-morbidities leading to high surgical or anaesthetic risks, poor renal function or a solitary kidney. Comparing percutaneous RFA to PN of small renal masses, RFA has been shown to be associated with less blood loss, smaller post-procedure drop in renal function and shorter length of hospital stay [[4], [5], [6]]. Medium term outcomes are also comparable with no statistical difference in 5 year tumour-related survival or local recurrence [[5], [6], [7], [8]].

The challenge with ablative therapies is determining whether a treatment is successful or not early in the course of treatment. Unlike surgery where pathological assessment of resection margins is possible, determination of complete ablation is more challenging. Early detection of persistent or recurrent tumour will change future management, particularly as the evidence for repeated, invasive surveillance biopsy is inconclusive [9]. Current practice is for initial cross-sectional imaging to be performed typically within 3 months post-procedure, and 3–6 monthly thereafter, the timing varying depending on institutional practice. Ablation is deemed successful if CT shows a hypoattenuating ablation zone with absence of contrast enhancement [10].

Features of persistent tumour that have been described include irregular, nodular enhancement >10 Hounsfield Units (HU) within the ablated area [10]. Whilst contrast-enhancement gives an indication of overall tissue contrast uptake (combination of both intra- and extra-vascular compartments), it is affected by contrast dose, administration rate and cardiac output [11]. Whilst the same is true of quantitative perfusion measurements the effects of these variables can be controlled and mitigated by longer imaging acquisition times and arterial input measurements [12]. It remains unclear whether measuring perfusion is advantageous over qualitative assessment and whether it has any impact on subsequent management. Thus we performed a systematic review of the available medical literature, focusing on whether perfusion imaging has a role as a response biomarker in the assessment of ablation therapies and whether perfusion imaging impacts on subsequent management.

## 2 Materials and methods

### 2.1 Data sources and search strategy

We identified primary studies investigating perfusion imaging after ablation of small renal masses from the PubMED database. We included both cryoablation and radiofrequency ablation, with post-procedural perfusion-CT or perfusion-MR or quantitative dual-energy CT.

The following combinations of search terms were applied to identify relevant studies:

(“kidney” OR “renal”) AND (“tumour” OR “tumor” OR “carcinoma” OR “lesion” OR “mass” OR “cancer”) AND (“RFA” OR “radiofrequency” OR “radio frequency” OR “cryotherapy” OR “cryotherapy” OR “cryoablation” OR “ablation” OR “ablative” OR “locoregional therapy”) AND (“CT” OR “MRI” OR “perfusion” OR “dual energy” OR "dual-energy" OR “response” OR “dynamic contrast enhanced CT “OR “dynamic contrast enhanced MRI “OR “DCE-CT” OR “DCE-MRI” OR “quantitative" OR “ASL” OR “arterial spin”).

Results were limited to human studies. No studies were excluded on the basis of language. Relevant systematic reviews were read in full to ensure appropriate studies had not been missed. The search was performed independently by 2 radiologists with any disagreements resolved by consensus.

### 2.2 Selection criteria

Electronic abstracts of identified studies were read and the following exclusion criteria applied: case reports, narrative reviews, letters/correspondence and conference abstracts were excluded as these would not contribute sufficient unbiased data able to answer our research question. An excluded study log recorded reasons for exclusions.

### 2.3 Data extraction

Data was extracted from the included full text articles and recorded on a database (Excel, Microsoft, Redmond WA, USA). For each article the publication details and primary characteristics (number of patients, age, size and histology of lesions, ablative technique used, imaging follow-up protocol, summary of findings) was recorded.

### 2.4 Meta-analysis

Whilst the intention was to perform a meta-analysis on the included data, this was precluded as only a small number of studies have been published with none sharing similar methodology.

## 3 Results

**Fig. 1:**
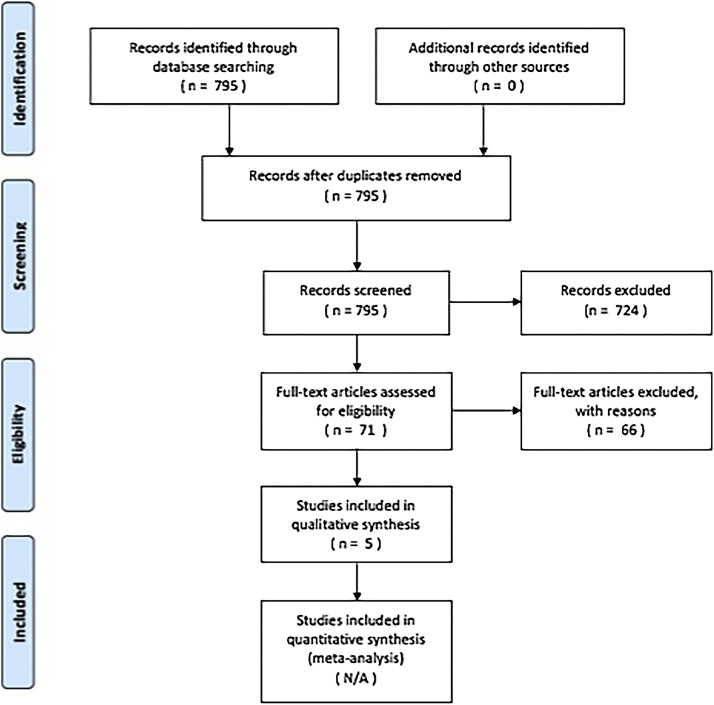
PRISMA (Preferred Reporting Items for Systematic reviews and Meta-Analyses) breakdown of systematic review search results.

PRISMA (Preferred Reporting Items for Systematic reviews and Meta-Analyses) guidelines for transparent reporting of systematic reviews were followed (Fig. 1).

### 3.1 Eligible studies

The initial search performed on 12 February 2018 yielded 795 articles. 724 articles were excluded following evaluation of abstracts. The remaining 71 articles were retrieved in full text and eventually 5/71 (7.0%) studies were included in the systematic review [[13], [14], [15], [16], [17]]. Of these, two studies investigated perfusion-MR following RFA [13,17] and one study each investigated perfusion-MR following cryotherapy [14], perfusion-CT following cryoablation [15], and dual-energy CT after RFA [16].

### 3.2 Patient population

Characteristics for the included studies are listed in Table 1. 110 patients were included with an individual study range of 10 to 47 patients. The patient age and renal mass sizes were reported differently between studies (range versus standard deviation). 4 studies were limited to RCC; one study included RCC and angiomyolipomata. 4 studies were prospective; 1 was retrospective. 4 studies were European in origin (Germany, Italy, 2 from UK); 1 was Korean. 7/110 patients had residual disease or recurrence following ablation.

**Table 1:** Characteristics of the included studies.

First author	Single OR Multi-centre	Accrual	Histology	Tumour size / cm	Ablation procedure	Imaging	Timing of imaging
Year							
Sample size							
Boss	Single	Prospective	RCC	1.6–3.9	MRI-guided RFA	DCE and ASL MRI	Before procedure
2006							Day 1
n = 10							6 weeks
Squillaci	Single	Prospective	RCC; angiomyolipoma	2.04 (1.5–2.9)	Laparoscopic cryoablation	Perfusion CT	6–8 months
2009							
n = 15							
Chapman	Single	Prospective	RCC	3.52 (SD ± 0.74)	CT-guided percutaneous cryoablation	DCE MRI	Before procedure
2013							1 month
n = 18							
Park	Single	Retrospective	RCC	2.0 (SD ± 0.9)	2 US-guided percutaneous RFA	Dual-energy CT	Mean 28 months (range 6–63 months)
2014					45 CT-guided percutaneous cryoablation		
n = 47							
Wah	Single	Prospective	RCC	2.5 (1.3–4.0)	CT-guided percutaneous RFA	DCE MRI	Before procedure
2018							1 month
n = 20							

### 3.3 Reported results

With the limited published evidence and varied methodologies in each of the five included papers, the reported results are summarised in Table 2 and described below.

**Table 2:** Results of the included studies.

First author	Changes in perfusion of ablation zone with successful therapy	No. of unsuccessful ablations	Detection of residual or recurrent disease	Effect on clinical management
Year				
Sample size				
Boss	73 ± 11% decrease in perfusion on Day 1; 84 ± 14% overall decrease by 6 Weeks.	2 / 10	Case 1 – 62% fall in perfusion on Day 1 but no further fall at 6 Weeks. Findings correspond to suspicious area on conventional MRI and subsequently biopsy-proven; Case 2 – 93% fall on Day 1; return to 53% of original perfusion at 12 months.	Not investigated
2006				
n = 10				
Squillaci	More gradual wash-in, lower peak amplitude, slower wash-out than normal renal cortex	1 / 15	Rapid early wash-in; plateau phase, and slow homogeneous wash-out compared with normal renal cortex	Not investigated
2009				
n = 15				
Chapman	mean 88.2% decrease in perfusion at 1 Month	0 / 18	N/A	Not investigated
2013				
n = 18				
Park	Peak contrast enhancement in late nephrogenic phase; Mean enhancement 12.1 ± 11.7 HU	4 / 47	Peak contrast enhancement in cortico-medullary phase; Mean enhancement 75.3 ± 40.9 HU	Not investigated
2014				
n = 47				
Wah	Mean 96% decrease in tumour perfusion following RFA	0 / 20 patients	N/A	Not investigated
2018		(0 / 21 lesions)		
n = 20				

### 3.4 Changes in perfusion with therapy

Boss et al. [13] compared perfusion-MR (both arterial-spin labeling and dynamic contrast enhanced MR) with T1W-gadolinium enhanced MR. Studies were performed before, 1 day and 6 weeks after MR-guided percutaneous RFA or RCCs. Prior to ablation, RCCs demonstrated “heterogeneous perfusion with zones of cystic tissue necrosis completely lacking perfusion” with a mean tumour perfusion rate of 167 ± 81 ml.100g^−1^. min^−1^. On the Day 1 imaging, the mean reduction was 73 ± 11%. In successful cases, the ablation zones demonstrated further decrease in perfusion between Day 1 and 6 weeks. The mean overall decrease from pre-treatment studies was 84 ± 14%. Wah et al. [17] also performed DCE-MRI before and 1 month after percutaneous RFA of RCCs. They found perfusion decreased significantly within the ablation zone. Interestingly, the degree of pre-ablation perfusion was correlated with the time taken for complete ablation.

Chapman et al. [14] performed DCE-MRI before and 1 month after cryoablation of RCCs in 18 patients. A surrogate measure of perfusion was calculated and then comparisons were made between the signal of the tumour, renal cortex and ablated area. Prior to treatment, mean perfusion within the tumour was 98.0 ml.100ml^−1^. min^−1^. On follow-up imaging, mean perfusion ablation zone perfusion was 11.6 ml.100ml^−1^. min^−1^. This is a decrease of 88.2% (P=<0.001). Only a single follow-up scan was performed on each patient.

Park et al. [16] used iodine-only images from dual-energy CT to quantify iodine-uptake and therefore to infer perfusion following RFA. No perfusion imaging was performed before treatment. Acquisitions were taken in the pre-contrast, corticomedullary and late nephrogenic phases. In successfully treated lesions, iodine uptake peaked in the late nephrogenic phase (mean increase 12.1 ± 11.7 HU). Statistical analysis was not performed.

Squillaci et al. [15] performed qualitative Perfusion-CT 6–8 months after laparoscopic cryoablation of small renal masses. No pre-therapy perfusion-CT imaging was performed. Perfusion curves for successfully treated ablation areas showed more gradual wash-in, lower peak amplitude, and slower washout compared with normal renal cortex.

### 3.5 Detection of residual or recurrent disease

In the study by Boss et al. [13] in 2/10 patients there was recurrent or residual disease identified using perfusion. In one patient, this manifested as a fall in ablation zone perfusion on Day 1 (174 ml.100g^−1^. min^−1^ to 66 ml.100g^−1^. min^−1^) but no further decrease in perfusion after 6 weeks (64 ml.100g^−1^. min^−1^). The findings on perfusion corresponded to a suspicious area on T2W and Gadolinium-enhanced T1W imaging, which was subsequently found to represent persistent RCC on biopsy. In a further patient, day 1 imaging confirmed a 93% fall in perfusion (321 ml.100g^−1^. min^−1^ to 25 ml.100g^−1^. min^−1^), however at the next study, 12 months later, high perfusion had returned (169 ml.100g^−1^. min^−1^). No histological confirmation was obtained as the imaging was conclusive for relapse.

Chapman et al. [14] and Wah et al. [17] found no patients (0/18 and 0/20, respectively) with residual or recurrent tumour on the 1 month imaging.

Park et al. [16] detected tumour recurrence in 4/47 patients. In this subgroup of patients, and unlike successfully ablated zones, iodine uptake peaked in the corticomedullary phase. Iodine uptake was also higher in areas of recurrent compared with controlled disease (mean 75.3 ± 40.9 HU v 7.4 ± 5.8 HU in the corticomedullary phase, and 52.1 ± 7.9 HU v 12.1 ± 11.7 HU in the late nephrogenic phase).

In the study by Squillaci et al. [15], 1/15 patients had residual tumour on standard post-contrast imaging, as demonstrated by enhancing nodularity at the tumour margin. On perfusion-CT, there was a characteristic perfusion curve; fast and early wash-in, followed by a plateau, and then progressive, uniform wash-out. Time-to-peak, wash-in rate, peak contrast enhancement, blood volume, blood flow, permeability, and mean transit time were all different between recurrent tumour and successfully ablated areas. Formal statistical analysis was not performed as there was only a single patient with recurrent tumour.

### 3.6 Effect on clinical management

No study assessed impact on initial management thus current data is insufficient to draw any conclusions on the impact of perfusion imaging on management.

## 4 Discussion

Our systematic review confirms a paucity of research in to the role of perfusion imaging as a response biomarker after ablation therapy in SRTs. To date, there are only 5 small studies, 1 retrospective, using a number of different imaging techniques following different ablation therapies. Pre-procedure perfusion imaging was only obtained for 3 of the studies. Perfusion in residual or recurrent disease has only been studied in 7 patients.

Ablative therapies partly owe their success to the greater tumour sensitivity to extremes of temperature compared to surrounding healthy tissue [18]. The mechanisms of action of ablative therapies at a cellular level have been previously described [19,20]. RFA uses a high-frequency alternating current to generate frictional heat. Within the central zone, immediately adjacent to the appliance tip, temperatures of 60-100**°**c cause direct and indirect cellular damage. The amount of damage is dependent on the total amount of thermal energy, rate of deposition, and thermal sensitivity of the tissues [18]. Above 60**°**c, there is rapid coagulative necrosis. The mechanisms for the secondary effects of hyperthermia are less understood but include changes to a combination of cell membrane permeability, mitochondrial function, enzymatic function, and DNA replication [21,22,20,23]. In the transition zone, there is reversible, sub-lethal heating due to thermal conduction. Following treatment, immune response is seen within this region [19].

Cryoablation uses liquefied gas that cools as it expands to cause extremes of cold. The expansion within the probe creates a heat sink, reducing temperatures to as low as -160**°**c. Cell death occurs when tissue temperature reaches between −20**°**c and −40**°**c [24]. This area of cooling needs to extend >1 cm beyond the tumour margin to cause complete ablation [25,26]. Cryoablation has several mechanisms of action. In areas of extreme cool, direct cell death is caused by cellular dehydration. Secondary tissue damage occurs due to vascular injury and ischaemia [27]. Cell apoptosis and a localised immune response are seen at the peripheries of the central zone [28,29].

Following tumour ablation size-based response criteria may not reflect the impact of therapy accurately as significant change in size may not occur. In the peri-procedural period following ablative therapy, ablation zones have demonstrated varied size responses, even including increased size [30]. Over time, there is involution in successfully treated areas. Following RFA there is approximately a 50% volume decrease after 2 years [31] and 75% decrease following cryoablation [32].

In the absence of surgery and with the lack of pathological confirmation of complete tumour destruction, the ability to identify incomplete ablation/recurrence early would allow further treatment at an earlier time-point. In addition, current post-procedure imaging can be inconclusive with a decision made to offer further ablation; a robust biomarker would be able to reassure that there is no recurrence without the need for invasive biopsy or undergoing unnecessary re-treatment. Current clinical practice is for pre- and post-contrast CT (or MR imaging where there is renal impairment). Particularly in the peri-procedural period, determination of successful tumour ablation can be challenging with standard imaging and biopsy due to high false-positive and false-negative rates [[33], [34], [35], [36]]. The limitations of qualitative assessment are well known. Contrast enhanced ultrasound has been suggested as an alternative to cross-sectional imaging although not all tumours are visible on B-mode ultrasound. In addition, the degree of enhancement is subjective [37]. Assessing perfusion quantitatively, rather than enhancement, is more robust taking into account differences in contrast agent administration, dose and cardiac output. As ablative therapy is increasingly utilised as a management option [38], it is essential that robust imaging biomarkers can be used to determine the success of treatment.

Our systematic review indicates that more evidence is still required, ideally through prospective studies. Studies have shown that successfully treated regions demonstrated reduced perfusion following therapy and sustained reduction in perfusion after 6–19 months, akin to observations in a case series investigating renal cell carcinoma perfusion-CT following anti-angiogenesis therapy [39], but there is still limited data on the perfusion characteristics of residual or recurrent tumour.

Residual disease or local recurrence has been reported in 5.6% and 4.2% of cases, respectively, following RFA treatment of T1a renal tumours [40]. It is important that future studies are powered to draw firm conclusions with respect to perfusion characteristics in residual or recurrent tumour. In our review of the literature no recurrences were detected just by perfusion imaging; the areas of abnormal perfusion corresponded to expected morphological appearances. Demonstrating any additional value over currently accepted contrast-enhanced CT or MRI is needed for it to be accepted as standard care.

In conclusion to date there is only limited evidence for perfusion imaging as a response assessment following ablative therapies. Further adequately powered prospective research is needed to determine whether there is an impact on clinical management for perfusion imaging to become part of standard of care.

## Conflict of interest

On behalf of all authors, the corresponding author states that there is no conflict of interest.
