# Corrigendum to “Is there a role for perfusion imaging in assessing treatment response following ablative therapy of small renal masses—A systematic review” [Eur. J. Radiol. Open 5 (2018) 102–107]

**DOI:** 10.1016/j.ejro.2019.07.001

**Published:** 2019-07-19

**Authors:** S.J. Withey, J. Gariani, K. Reddy, D. Prezzi, C. Kelly-Morland, S. Ilyas, A. Adam, V. Goh

**Affiliations:** a Department of Radiology, Guy’s and St. Thomas’ NHS Foundation Trust, London, United Kingdom; b Cancer Imaging, Division of Imaging Sciences and Biomedical Engineering, King’s College London, United Kingdom

The authors regret “that the acknowledgement in the previous version of the article was not complete. The revised acknowledgement shall read as follows: The authors acknowledge the support from the Department of Health via the National Institute for Health Research Comprehensive Biomedical Research Centre award to Guy’s & St. Thomas’ NHS Foundation Trust in partnership with King’s College London and King’s College Hospital NHS Foundation Trust; Wellcome/EPSRC Centre for Medical Engineering [WT 203148/Z/16/Z] that supports V. Goh; and from the King's College London/University College London Comprehensive Cancer Imaging Centre funded by Cancer Research UK and Engineering and Physical Sciences Research Council in association with the Medical Research Council and Department of Health”.

The authors would like to apologise for any inconvenience caused.

